# Complete Coding Sequence of a Pasivirus Found in Swedish Pigs

**DOI:** 10.1128/MRA.00747-20

**Published:** 2020-09-24

**Authors:** Giorgi Metreveli, Mikael Berg, Anne-Lie Blomström

**Affiliations:** a Department of Biomedical Sciences and Veterinary Public Health, Swedish University of Agricultural Sciences, Uppsala, Sweden; KU Leuven

## Abstract

Here, we report the complete coding sequence of a pasivirus found in the tonsil of a conventionally reared pig from a herd with respiratory disease in Sweden. The genome displays 75% to 87% and 81% to 94% nucleotide and amino acid sequence identity, respectively, to genomes of pasiviruses from other parts of the world.

## ANNOUNCEMENT

Pasiviruses (genus *Pasivirus*, family *Picornaviridae*) are believed to have swine as their natural host. Similar to other members of the family *Picornaviridae*, they have a positive-sense single-stranded RNA genome encoding a single polyprotein (1). The first member of genus *Pasivirus* was discovered in healthy piglets in France in 2012 through the use of high-throughput sequencing (2). Since that first discovery, pasiviruses have been found in other parts of the world, such as China, Hungary, the United States, Germany, and Romania (3–6). In a recent metagenomic study, we showed that pasivirus is also present in Sweden (7). As only a short region (1,481 nucleotides [nt]) was obtained through the metagenomic analysis, we have now analyzed these samples further and report here the complete coding sequence for a pasivirus found in Swedish pigs.

A viral metagenomic approach was used to investigate the viral composition of tonsil samples from eight conventionally reared Swedish pigs from a farrow-to-finish herd that has a history of a high prevalence of respiratory lesions registered in the respiratory tract of the pigs at slaughter. Through this analysis, a longer pasivirus contig was identified in one of the samples (7). In short, the tonsil tissue was homogenized in sterile phosphate-buffered saline (PBS) using Precellys CK14 tubes prior to filtration (0.45 μM) and DNase I treatment. RNA was extracted using a GeneJET RNA extraction kit, and DNA was extracted using a GeneJET genomic DNA purification kit. Prior to sequencing, the RNA was reverse transcribed into cDNA and randomly amplified by single-primer isothermal amplification (SPIA) using the Ovation transcriptome sequencing (RNA-seq) V2 kit. The DNA was also randomly amplified by random PCR (rPCR) as previously described (8, 9). The products were sequenced at SciLifeLab/Genome Center (Uppsala, Sweden) using the Ion S5 XL system, the Ion Plus fragment library kit for the AB Library Builder system was used to build the libraries, and the libraries were loaded onto a 530 chip. The raw reads (1,600,262; average length, 243 nt) were trimmed based on length (≥50 nt) and quality (Q ≥ 20) (number of reads after trimming, 1,419,391; average length, 190 nt). The *de novo* assembly tool in CLC Genomic workbench (v12) was used to assemble the reads using the default parameters. BLASTX through Diamond (v0.8.26) was used to annotate the reads. Although the majority of the viral reads in this tonsil belonged to teschovirus, additional viruses were identified, such as adenovirus and parvovirus, as well as pasivirus. The 125 pasivirus reads did not cover the complete genome, but assembly of the reads yielded a 1,481-nt-long contig (7). As pasivirus has not been detected in Sweden before and few pasivirus genome sequences are publicly available, in this study, we sequenced the complete coding region of the detected pasivirus. The mentioned contig and additional pasivirus reads found in the data set were used to design primers (Table 1) to cover the complete coding sequence of the identified pasivirus.

**TABLE 1:** Primers used to amplify the complete coding sequence of the investigated pasivirus

Primer name	Primer sequence (5′–3′)
Pasi_1F	TAGGGTGTGGGCCTGC
Pasi_1R	CCACCCACACTCAGAAGAATC
Pasi_2F	CTGGAATAGTAAAGATTTGATATTGGCTAC
Pasi_2R	CTGACCGCTACTCACATCC
Pasi_3F	TGCACACAATAATTCTTCTGCTAATG
Pasi_3R	CGAGCACTCAAAGACATTGC
Pasi_4F	TAAACAGTTAATTAAAGATACGTTGGGATC
Pasi_4R	CTGTTTAACAATATCAGTAATAAGATCATTAACAG
Pasi_5F	AATTATACCGTTAGGGCACGC
Pasi_5R	AAGGTTGGTGCACACGTAC
Pasi_6F	CTTACACGTGGCTGGTAATGG
Pasi_6R	TGACCACAAACCTAGGAAAGC

**FIG 1:**
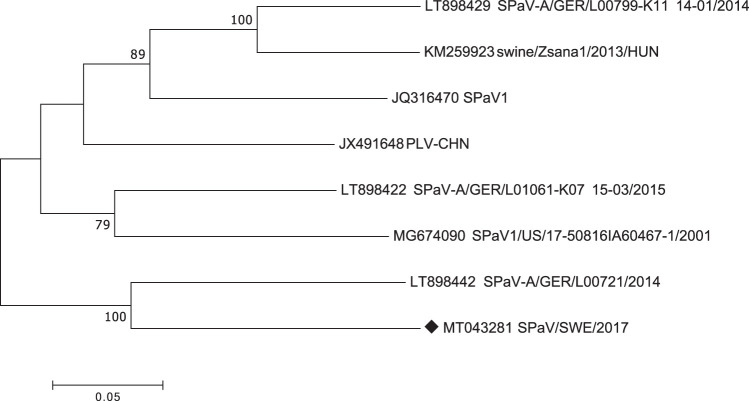
Phylogenetic analysis of the complete coding sequence of porcine pasiviruses. The evolutionary history was inferred by using the maximum likelihood method based on the Tamura-Nei model. There were a total of 6,388 positions in the final data set. Evolutionary analyses were conducted in MEGA7 (10). The sequence from our study is marked with a black diamond.

PCR was performed using the Platinum SuperFi DNA polymerase; the PCR products were purified using a GeneJET PCR purification kit and sent to Macrogen for Sanger sequencing. The quality check and the assembly of the Sanger sequences were performed using SeqMan (Lasergene v9; DNAStar). A 6,402-bp-long nucleotide sequence, with a GC content of 42%, covering the complete coding region of the pasivirus genome, was obtained. The sequence for isolate SPaV-A/SWE/2017 has been submitted to GenBank (accession number MT043281.1). ClustalW and pairwise analysis showed that the obtained genome has a 75% to 87% nucleotide identity to the currently available full-length genomes at NCBI. The closest similarity was to a German isolate (LT898422) sampled in 2014. The reported nucleotide sequence codes for a 2,134-amino-acid (aa)-long polyprotein, and the sequence identity on an amino acid level is 81% to 94% to other pasiviruses available at NCBI. Phylogenetic analysis of the Swedish isolate together with the seven available complete genomes at NCBI was performed with MEGA7 (10) using maximum likelihood and a bootstrap value of 1,000. In this analysis, the Swedish isolate grouped with the previously mentioned German isolate (Fig. 1).

The polyprotein amino acid sequence was analyzed further using InterPro, and several conserved domains were identified, as follows: rhv-like/Picornavirus coat protein (aa position 105 to 487), helicase of super family 3 (aa 1152 to 1317), core protein P3A (aa 1380 to 1462), picornavirus peptidase C3A/C3B (aa 1529 to 1661), and RNA-directed RNA polymerase C-terminal domain (aa 1693 to 2119).

In summary, we report the complete coding sequence of a pasivirus infecting pigs in Sweden. At present, few viral genome sequences of this virus are publicly available, and further studies are needed to determine the evolution of these viruses and what effect they may have on porcine health.

### Data availability.

The genome sequence has been deposited in GenBank under the accession number MT043281.1, and the data set containing the raw reads has been deposited under the BioSample accession number SAMN08969270.

## References

[1] Zell R, Delwart E, Gorbalenya AE, Hovi T, King AMQ, Knowles NJ, Lindberg AM, Pallansch MA, Palmenberg AC, Reuter G, Simmonds P, Skern T, Stanway G, Yamashita T, ICTV Report Consortium 2017 ICTV virus taxonomy profile: Picornaviridae. J Gen Virol 98:2421–2422. doi:10.1099/jgv.0.000911. 28884666 PMC5725991

[2] Sauvage V, Ar Gouilh M, Cheval J, Muth E, Pariente K, Burguiere A, Caro V, Manuguerra JC, Eloit M 2012 A member of a new Picornaviridae genus is shed in pig feces. J Virol 86:10036–10046. doi:10.1128/JVI.00046-12. 22787214 PMC3446573

[3] Guo B, Kim H, Zheng Y, Shen H, Pogranichniy RM, Schwartz KJ, Li G, Yoon KJ 2018 Genomic sequence of a swine pasivirus type 1 strain identified in U.S. swine. Genome Announc 6:e01569-17. doi:10.1128/genomeA.01569-17. 29439048 PMC5805886

[4] Hanke D, Pohlmann A, Sauter-Louis C, Hoper D, Stadler J, Ritzmann M, Steinrigl A, Schwarz BA, Akimkin V, Fux R, Blome S, Beer M 2017 Porcine epidemic diarrhea in Europe: in-detail analyses of disease dynamics and molecular epidemiology. Viruses 9:177. doi:10.3390/v9070177. PMC5537669 28684708

[5] Yu J-M, Li X-Y, Ao Y-Y, Li L-L, Liu N, Li J-S, Duan Z-J 2013 Identification of a novel picornavirus in healthy piglets and seroepidemiological evidence of its presence in humans. PLoS One 8:e70137. doi:10.1371/journal.pone.0070137. 23936384 PMC3735577

[6] Zaulet M, Petrovan V, Birladeanu AM, Stoian AMM, Kevorkian SEM, Nichita C, Eloit M, Buburuzan L 2017 Identification and prevalence of swine pasivirus 1 in eastern Romanian pig farms. J Vet Diagn Invest 29:305–311. doi:10.1177/1040638717696044. 28363267

[7] Blomstrom AL, Ye X, Fossum C, Wallgren P, Berg M 2018 Characterisation of the virome of tonsils from conventional pigs and from specific pathogen-free pigs. Viruses 10:382. doi:10.3390/v10070382. PMC6071052 30036964

[8] Allander T, Tammi MT, Eriksson M, Bjerkner A, Tiveljung-Lindell A, Andersson B 2005 Cloning of a human parvovirus by molecular screening of respiratory tract samples. Proc Natl Acad Sci U S A 102:12891–12896. doi:10.1073/pnas.0504666102. 16118271 PMC1200281

[9] Blomström AL, Widen F, Hammer AS, Belak S, Berg M 2010 Detection of a novel astrovirus in brain tissue of mink suffering from shaking mink syndrome by use of viral metagenomics. J Clin Microbiol 48:4392–4396. doi:10.1128/JCM.01040-10. 20926705 PMC3008476

[10] Kumar S, Stecher G, Tamura K 2016 MEGA7: Molecular Evolutionary Genetics Analysis version 7.0 for bigger datasets. Mol Biol Evol 33:1870–1874. doi:10.1093/molbev/msw054. 27004904 PMC8210823

